# Gross Negligence in Bank Payments Law

**DOI:** 10.1093/ojls/gqag002

**Published:** 2026-02-02

**Authors:** Jo Braithwaite

**Keywords:** banks, payment, gross negligence, payment services, fraud, Financial Ombudsman Service

## Abstract

The law’s allocation of responsibility for disputed bank payments is important not only for banks and customers, but also, given the volume and value of payments, for the economy more broadly. This article examines loss allocation under common law, EU–UK regulation and the UK’s ‘world-first’ scheme for ‘authorised push payment’ frauds, showing that the gross negligence standard of customer conduct is a common feature. Indeed, this ‘slippery concept’ sits at critical junctures, providing, in practice, the most relevant limitation to a bank’s liability to refund payments. The article analyses what this standard means in English common law and in its regulatory contexts, and highlights the important connection between the two. It then draws upon decisions by the UK’s Financial Ombudsman Service, amongst other sources, to explore how evaluating bank customer conduct works in practice, concluding that it is time for regulators to rethink the status quo.

## 1. Introduction

In what circumstances is a bank account holder entitled to have a payment reimbursed by their bank? This question has important implications not only for banks and their customers, but also, given the volume and value of bank payments, for the economy more broadly. In the UK domestic market alone, in 2023 there were 4.9 billion ‘remote banking’ payments, 4.8 billion direct debits and 24.5 billion debit card payments.^1^ In addition, the payment system CHAPS, which is typically used for transfers between participants in the wholesale markets, accounted for £91.5 trillion of payments (88% of UK payments by value).^2^

This article examines the loss allocation regimes that have evolved in respect of several different categories of bank payments. While these regimes vary in significant ways, the article shows that the gross negligence standard of conduct is a common feature. Indeed, what an English judge recently referred to as this ‘slippery concept’^3^ sits at critical junctures across payments law, providing what is, in practice, the most relevant limitation to a bank’s liability to refund disputed payments.^4^ The gross negligence standard features, for instance, in widely used exculpatory terms in bank–customer contracts, as well as in important EU–UK legislation allocating losses for unauthorised payments.^5^ Most recently, this standard has also been deployed in the UK’s ‘world-first’ reimbursement scheme for ‘authorised push payment’ fraud, as part of a long definition of a ‘Consumer Standard of Caution’.^6^

There are several reasons why examining the gross negligence standard in bank payments law matters and is timely. First, since the pandemic, greater attention is being paid to frauds involving the diverse payment methods now used by bank customers, representing losses of some £1.17 billion in 2024, across 3.31 million confirmed cases.^7^ As the article shows, gross negligence is used across different areas of bank payments law as a means of limiting default rules which otherwise protect customers from losses. Consequently, this standard, its meaning and its application to payment disputes have all become increasingly significant in practice, which issues this article assesses from a variety of perspectives. Secondly, the EU and UK legal regimes which incorporate this standard of conduct and are evaluated in this article are all at various stages of review, meaning that this is a moment of unusual potential to re-evaluate the status quo.^8^ Thirdly, this analysis also contributes to a better understanding of the law of bank payments generally; indeed, despite its significance to the global financial markets, the real economy and the exercise of important social rights, this area of law remains under-researched in the scholarly literature.

The article proceeds in four main parts. The following section (section 2) explains the relevant legal frameworks in bank payments law, focusing on the ‘gross negligence’ standard of conduct as it features across these different regimes. Section 3 analyses what this standard means both in English common law and in its regulatory contexts, and highlights the important connection between the two. Section 4 draws upon published decisions by the UK’s Financial Ombudsman Service, amongst other sources, to explore how evaluating bank customer conduct against this standard works in practice in the UK. The final main section, section 5, sets out the implications of the analysis and argues that it is time for regulators to rethink how to delineate liability in this important context, in order to promote fairness, transparency and trust in the bank–customer relationship.

## 2. How Is the Gross Negligence Standard Relevant to Payments?

The starting point for this article is that the ‘gross negligence’ standard is central to the various legal regimes allocating liability for payments. Both common law and regulation are relevant to the allocation of losses in the case of disputed bank payments, depending on factors such as the type of account holder and the currency of the transaction, while there are different regulatory rules which apply to authorised and unauthorised payments from bank accounts. This section considers how the gross negligence standard is used to delineate liability in each of these legal regimes, demonstrates how regulation builds upon long-standing common law in this respect and explores the trade-offs associated with this standard.

### A. Common Law

Since foundational authority in the 19th century, English common law has provided that the account-holding relationship is one of contract, where, if an account is in credit, the customer is an unsecured creditor of the bank.^9^ This authority underpins the legal aspects of making a payment from a bank account.

The common law of bank payments was central to the Supreme Court’s recent analysis in *Philipp* v *Barclays Bank*^10^ because the payments in question fell outside the scope of the regulations considered below (as they were sent overseas).^11^ As *Philipp* set out, a bank payment must be authorised by the customer in the manner provided for in the account-holding contract, following which the bank has a ‘strict’ duty to follow the customer’s instructions promptly. Conversely, a payment from an account which is not duly authorised by the customer (ie an unauthorised payment) must be refunded by the bank, on the basis that making such a payment is a breach of contract.^12^ The Supreme Court in *Philipp* went on to decide that a bank will have a duty to make inquiries to verify a payment instruction where the instruction is from an agent purporting to act on behalf of a customer and the bank is ‘on inquiry’ that the instruction might be an attempt to defraud the customer, but that this duty will not apply when valid instructions are received from an individual acting on her own behalf.^13^

In common law, customers’ duties during the payment process are limited to the so-called ‘Macmillan’ and ‘Greenwood’ duties.^14^  *Macmillan* held that the customer has a duty to provide clear written instructions so as not to mislead the bank or facilitate forgery;^15^  *Greenwood*, that the customer has a duty to inform the bank within a reasonable time when they know of a fraud on their account.^16^ Breach of either duty leads to the customer bearing the loss of the unauthorised payment from their account. Absent express provision in the account-holding terms, banks’ attempts to broaden customers’ duties relating to unauthorised payments, for example, so that a customer would be responsible for checking bank statements for fraud, have failed.^17^ As the Privy Council observed in *Tai Hing*, banks could impose extra duties on customers by including them in the express terms of the account-holding contract.^18^ If express terms were to be included to adjust the common law default by reference to the customer’s ‘gross negligence’, such terms would, in turn, be interpreted in line with the authorities discussed in section 3 below.

Looking to the bank’s own conduct, mandate letters and other agreements used to document the account-holding relationship between banks and sophisticated clients routinely include exculpatory clauses limiting the bank’s liability to that arising from fraud, wilful default or gross negligence. Indeed, one of the most significant recent decisions on the meaning of gross negligence in English civil law involved a sophisticated customer’s allegations the bank had acted with gross negligence during the payments process, in the context of an exculpatory term of this kind.^19^ As we shall see, because there is no statutory definition of gross negligence in payments regulation, such decisions on the meaning of the standard in sophisticated parties’ contracts currently inform the meaning of a *customer’s ‘*gross negligence’ in the regulatory context.

### B. EU–UK Legislation

In practice, the regulation of the account-holding relationship has evolved dramatically from the *Foley* era. Today, layers of legislation, express terms and conditions, codes of practice and soft law wrap around the common law ‘core’, though the common law remains directly relevant to certain payments, as highlighted at the end of this subsection. An early milestone in this process was the introduction of the British Banking Association’s Banking Code, the first edition of which came into effect on 16 March 1992^20^ following recommendations in the Jack Committee’s 1988 report.^21^ The loss allocation rules for payments which we know today can be traced back to this groundbreaking Banking Code and it is therefore important to understand how and why its provisions were introduced.

Prior to the introduction of the 1992 Banking Code, the bank–customer relationship was still arranged on the traditional, largely common law basis; as the Jack Committee put it, ‘In this area implied contract still, for the most part, holds sway’,^22^ while regulation of this relationship was a matter of ‘somewhat ancient’ case law^23^ and a handful of statutes.^24^ Over its two-year review, the Jack Committee considered this ‘patchy framework’ governing banking services against ‘four fundamental objectives’: fairness and transparency in the banker–customer relationship; ‘maintenance of confidence in the security of the banking system’; the promotion of efficiency; and the ‘preservation and consolidation of the banker’s duty of confidentiality’.^25^ Finding that traditional arrangements were insufficient, especially in light of developments in technology,^26^ the Jack Committee Report made a series of recommendations designed to improve standards. These recommendations included that the banking industry should formulate a non-statutory Code of Banking Practice, which would deal, *inter alia*, with ‘the apportionment of loss arising from a disputed [electronic funds transfer]’.^27^ The Report made it clear that this was a pivotal moment for payments law because:

Historically, the development of standards of best banking practice has been the sole prerogative of the banks. It has been argued in this Report that that approach is no longer entirely appropriate … While banks must continue to have a major say in the development of standards of best practice, there is also a legitimate public interest in those standards …^28^

The groundbreaking 1992 ‘Good Banking: Code of Practice’ emerged from this recommendation.^29^ Although the Code was voluntary for banks and building societies, it represented a significant departure from the status quo, and it generated considerable controversy. Indeed, contemporary reports suggest that it took longer than expected for draft versions to be consulted upon because consumer groups responded with ‘severe criticism’ and put forward ‘some significant revisions’.^30^ Most notably for this purpose, the draft Code sent out for consultation included the provision that customers may be liable for losses from lost or stolen bank cards, even once notified to the card issuer, ‘if they have acted fraudulently or *negligently*’.^31^ However, contemporary reports suggest that consumer groups objected to this broad carve-out.^32^ In the end, this limitation on liability was revised to refer to customers’ ‘gross negligence’ in the final version, while the burden of proof was also shifted from the position in the draft Code, so that it was now the card issuer’s burden to prove that the customer so acted.^33^ As we shall see later in the article, this calibration of liability was a significant outcome from the Jack Committee’s work.

European law makers have also long been active in this area. In 1988, for example, the EC issued a non-binding Recommendation which included the provision that liability for lost, stolen or copied payment devices should lie with card issuers, apart from in the case of the card holder’s fraud or ‘extreme negligence’, as noted and discussed by the Jack Committee Report.^34^ In terms of hard law, there have been two EU Payment Services Directives so far, the first from 2007 (PSD1)^35^ and the second from 2015 (PSD2),^36^ with the latter repealing the former. A third PSD and a new EU Regulation are currently under discussion, billed as an evolution rather than a fundamental change.^37^ The two Payment Services Directives were implemented into UK law by the Payment Services Regulations 2009^38^ and 2017^39^ respectively, and the latter remain in force post-Brexit.^40^ While the increasing complexity of the sector is reflected in the provisions of PSD2 when compared to its predecessor,^41^ the liability regimes for unauthorised payment transactions are almost the same in both versions, and therefore in the UK rules.^42^

The PSD’s coverage is broad though there remain some important limits, for example, it does not cover payments in cash.^43^ The PSD2 regime applies to ‘payment transactions in the currency of a Member State where both the payer’s payment service provider and the payee’s payment service provider are, or the sole payment service provider in the payment transaction is, located within the Union’ as well as to payment transactions in other currencies in certain circumstances.^44^ The term ‘payment service provider’ (PSP) is defined broadly, and includes credit institutions, electronic money institutions, ‘post office giro institutions’ and payment institutions, while Title IV, of most relevance to this discussion, covers transfers both in bank money and in electronic money.^45^

The varieties of unauthorised bank payments which fall within the EU and UK legislative regimes are therefore broad, including the billions of routine bank payments made each year by consumers within the UK. Nonetheless, in the UK, the common law remains relevant, for two reasons. First, where a particular bank transfer falls outside the scope of this legislation, for example, where sophisticated parties take advantage of their ability to contract out,^46^ the default position will be that provided by the common law. Secondly, even where the PSD does apply, it interacts with underlying national law. For the most part, this regime expressly excludes national law in favour of its own provisions, as seen most strongly in the ‘full harmonisation’ provision of the PSD as upheld by the European Court of Justice (ECJ).^47^ In a few places, however, the PSD expressly *includes* national law, folding it into the statutory regime. Both types of interaction are found in the PSD’s provisions for unauthorised payments, considered next.

### C. Unauthorised Payments in EU–UK Legislation

As in English common law, the default position under Title IV of the PSD is that banks must execute duly authorised payments,^48^ and will be liable to refund customers for any unauthorised payments.^49^ The PSD regime, however, imposes further pro-customer rules,^50^ and is more detailed and technically precise than the common law. For example, it specifies that customers must report unauthorised transactions without undue delay and no later than 13 months from the debit date in order to be eligible for reimbursement.^51^

Article 74 of PSD2 sets out various circumstances when this default refund rule for unauthorised payments will be qualified or disapplied altogether. The UK Payment Services Regulations (UK PSR) implement this provision in near-identical terms, which are then tracked in major High Street banks’ terms and conditions.^52^ In terms of the qualification, a customer may (in the earlier version of the regime, ‘shall’) be liable for a specified excess of up to €50 (previously, €150) if the unauthorised transactions were ‘resulting from the use of a lost or stolen payment instrument or from the misappropriation of a payment instrument’.^53^

Article 74 then provides two grounds upon which the default rule will be disapplied so that the customer shall be entirely liable for an unauthorised transaction.^54^ The first is if the customer has acted fraudulently; and the second is if the customer has, with ‘intent or gross negligence’, breached any of their obligations as set out in article 69 in relation to their ‘payment instrument’^55^ or ‘personalised security credentials’.^56^ These article 69 obligations extend to using the payment instrument according to the terms on which it had been issued; reporting its loss, theft, misappropriation or unauthorised use to the bank or the bank’s nominated entity without undue delay; and taking ‘all reasonable steps to keep the security credentials that go with the instrument safe.^57^ As discussed further in section 3 below, because it is onerous in practice to prove an allegation of fraud or ‘intent’, ‘gross negligence’ has a pivotal role in determining liability for unauthorised transactions under the PSD regime.

Perhaps surprisingly, given the importance that it bears in this regime, gross negligence is not expressly defined for the purposes of this legislation, at either the EU or UK level. In the PSD, evaluating the ‘evidence and degree of alleged negligence’ is expressly reserved for national law.^58^ While PSD1 did not address further what gross negligence meant in this context, a new Recital added to PSD2 stated that

gross negligence should mean more than mere negligence, involving conduct exhibiting a significant degree of carelessness; for example, keeping the credentials used to authorise a payment transaction beside the payment instrument in a format that is open and easily detectable by third parties.^59^

Unlike in other areas where EU legislation has reserved the meaning of private law concepts for national law, there is no legislative provision in the UK defining the term for this purpose. A contrasting approach is seen in the context of the EU’s Credit Rating Agencies Regulation, passed in 2009 and amended in 2013, so almost in parallel with the two PSDs. The Regulation provides that an investor or issuer is able to bring a civil claim against a credit rating agency where the latter has committed any of a list of infringements ‘intentionally or with gross negligence’.^60^ As with the PSD, the Regulation confirmed that private law terms including ‘gross negligence’ ‘shall be interpreted and applied in accordance with the applicable national law’.^61^ Unlike the PSD regime, however, UK secondary legislation followed, which expressly defines this and other legal concepts reserved for national law for the purpose of the Regulation.^62^

There is, however, some non-statutory guidance on the meaning of ‘gross negligence’ in the PSD context. The UK financial regulator, the Financial Conduct Authority (FCA), references the issue in its overall guidance on the PSD regime, though this largely confirms the key points in the PSD2 Recital. On process, therefore, the FCA’s ‘Approach’ document reiterates that the bank has the burden of proof and that there must be ‘sufficient evidence’ of gross negligence. It states that ‘Each case must be assessed on its merits’ and ‘we interpret “gross negligence” to be a higher standard of negligence than the standard of negligence under common law. The customer needs to have shown a very significant degree of carelessness.’^63^ However, detail about what this means for parties assessing customers’ conduct is lacking while, to the extent that this guidance paraphrases the PSD2’s Recital which, in turn, states that the ‘evidence and degree of alleged negligence should generally be evaluated according to national law’, the effect is circular.

### D. ‘Authorised Push Payment’ Scams

The framework discussed thus far covers unauthorised payments. Recently, separate UK regulation has been introduced to address *authorised* push payment (APP) frauds, which have proliferated in recent years. APP frauds involve a victim being tricked into making a payment in the course of a scam, while thinking they are involved in a legitimate transfer.^64^ These transactions fall outside the regime for unauthorised payments outlined in the preceding subsections, so, assuming that the fraudster is impossible to trace and/or impractical to sue, victims were traditionally left without legal recourse. This default position was confirmed by the UK Supreme Court in *Philipp*.^65^ The UK’s new mandatory reimbursement schemes, covering APP scam payments made using two of the major payment systems for UK domestic payments, CHAPS^66^ and the Faster Payment Scheme (FPS),^67^ were implemented shortly afterwards, applying to payments made on or after 7 October 2024. Unlike the PSD regime, discussed above, these rules represent a radical departure from the underlying common law position for payments in scope, in that payment service providers are now required to refund FPS and CHAPS payments (up to £85,000), whereas they would not be liable to do so in common law. The design and coverage of these ‘world-first’ schemes have been discussed elsewhere;^68^ the purpose of this subsection is to highlight the important, but over-complicated, way in which the concept of customer gross negligence has been deployed in this context.

Within this novel reimbursement regime, the limits to the mandatory refund rule are defined by reference to a customer’s fraud^69^ or gross negligence, as observed in the PSD context. However, in this new scheme, the latter standard is incorporated within a wide-ranging list of expectations for customer conduct, which is defined as the ‘Consumer Standard of Caution’. Specifically, an APP fraud victim will not be entitled to reimbursement if ‘as a result of gross negligence, [they have] not complied with one or more of the following [four] standards’, which refer to the customer ‘having regard’ to an intervention during their payment process by their payment service provider or an official agency such as the police; prompt reporting by the customer; customers responding to ‘reasonable and proportionate’ requests by their payment service provider (PSP) for information while the PSP is investigating a claim as provided for in the scheme; and consenting to the claim being reported to the police or reporting directly to an official agency.^70^

While there is of course good reason to require customers to co-operate with crime agencies in relation to APP fraud, it is not clear what overlaying this list of duties with a customer gross negligence standard achieves over simpler alternatives. For example, rather than framing ‘consent’ to reporting as a ‘standard’, breach of which as the result of ‘gross negligence’ limits the customer’s right to a refund, it would be simpler to impose a requirement that the customer provides this consent as a condition of reimbursement, subject to any necessary exceptions.^71^

Notably, unlike in the PSD regime, the APP scheme also expressly provides that the Consumer Standard of Caution will not apply at all if the case involves a ‘Vulnerable Customer’, and ‘this had a material impact on their ability to protect themselves from the scam’.^72^ The scheme adopts the FCA’s definition of a ‘Vulnerable Customer’ as ‘someone who, due to their personal circumstances, is especially susceptible to harm—particularly when a firm is not acting with appropriate levels of care’.^73^ Given that this kind of fraud, by definition, depends on trickery and manipulation, however, it seems likely that many more victims of APP fraud will be categorised as vulnerable than in the context of other types of payment disputes. Indeed, the voluntary code which pre-dated the APP reimbursement scheme included the important starting point that ‘All Customers can be vulnerable to APP scams and vulnerability is dynamic’.^74^ It follows that if this analysis is borne out in practice, as the discussion of recent Ombudsman decisions later in the article also suggests will be the case, the ‘Consumer Standard of Caution’ risks becoming sidelined, including with its expectations around crime reporting and co-operation with authorities. This is another reason for setting out such requirements separately.

There is some guidance on the customer conduct aspect of the APP scheme: what is referred to as the Consumer Standard of Caution ‘Exception’ (ie where a customer fails ‘as a result of gross negligence’ to meet one or four of the duties) is the subject of a PSR Guidance document.^75^ In important respects, the Guidance echoes the position in the EU–UK regime for unauthorised payments, in that it confirms that the burden of proof for customer gross negligence lies with the PSP; states that PSPs’ contracts cannot increase the required standards of customer conduct from those set out in the Consumer Standard of Caution; and further states that each case must be ‘assessed on its individual merits’. More substantively, while not referencing legal authorities, the Guidance makes the point that gross negligence in this context means ‘a higher standard than the standard of negligence under common law. The consumer needs to have shown a ‘significant degree of carelessness’’, which copies almost exactly the definition in the FCA Guidance on the PSD.^76^

Overall, however, the APP provisions amount to a complicated loss allocation provision with several moving parts. As with the PSD, customer gross negligence is not expressly defined for this purpose, while regulatory guidance follows the same lines in both areas; unlike the PSD, however, this standard will automatically not apply where a customer is vulnerable, which description may, arguably, apply to a large proportion of genuine victims of an APP scam, while also potentially applying in an uneven way to similar cases involving authorised and unauthorised transactions.

In keeping with the experimental nature of this scheme overall, the regulator has made a commitment that it will be reviewed within two years.^77^ In the meantime, this groundbreaking UK model has already been rejected by the EU, in favour of much more limited provisions around APP fraud in the proposed PSD3. The Commission’s proposed reforms in this area seek to protect customers’ trust in the banking system specifically.^78^ The proposed rules therefore limit a payment service provider’s liability to refunding only those APP losses due to ‘impersonation fraud’, where this involves ‘a third party pretending to be an employee of the consumer’s payment service provider’, on the basis that these ‘spoofing’ scams ‘affect the good repute of bank’. Reimbursement would be for the ‘full amount’ of losses, once again unless the payer has acted with fraud or gross negligence, or fails to report as required.^79^

This EU proposal has already generated considerable debate. The European Central Bank has noted that under the proposed rules, providers ‘are only liable proportionately to their level of control for this type of fraud’;^80^ however, the approach has been criticised as too narrow both by the European Economic and Social Committee^81^ and by the majority of the authors of a scholarly paper drawing on comparative insights from across several Member States, who argue that the right to reimbursement should arise from an abuse of trust in the payment system more broadly.^82^ The same paper also suggests that one consequence of new rules being introduced on these lines will be that the evaluation of customer ‘gross negligence’ will become increasingly relevant and nuanced, as considered further below.^83^

## 3. What Is the ‘Gross Negligence’ Standard in English Law?

So far, the discussion has explained how the standard of gross negligence plays a central role in loss allocation regimes across bank payments law, either because of the parties’ express terms or because a disputed payment falls under EU–UK payments regulation or the UK’s new reimbursement scheme for APP frauds. We have also seen that EU payments regulation references national law on the meaning of this standard, but also that the UK regulations do not define the standard expressly, though there is some FCA guidance on the issue.^84^ The result is that, in the UK, the meaning and application of the gross negligence standard in the bank payments context will be underpinned by common law, whether a particular scenario is governed by contract or by payments regulation. Given the nature of this legal concept, as this section explores, this regulatory design is not without its problems.

### A. Preliminary Questions

While in English tort law, there is only one standard of care at the liability stage,^85^ as we have seen, ‘gross negligence’ routinely features across various common law and legislative contexts, while it also serves as the fault standard in the controversial criminal offence of ‘gross negligent manslaughter’.^86^ Applying the ‘gross negligence’ standard therefore inevitably involves addressing preliminary, context-specific questions about the meaning of the term in that particular setting. In some contexts, for example, the term may simply provide a blank space on which parties project their own definition;^87^ elsewhere it may be importing ‘fraud-adjacent’ expectations around minimum standards of behaviour.^88^

This exercise raises the question of whether there is a requirement for a defendant to have acted with intent or subjective awareness for their act or omission to qualify as gross negligence. This issue has proved divisive. David Kershaw has shown that what he calls the ‘akin-to-fraud’ concept of gross negligence^89^ was a ‘formative idea in US law on both bailee and directorial standards of care’.^90^ In New York law, for example, gross negligence ‘differs in kind, not only degree, from claims of ordinary negligence’,^91^ while, tellingly, it is not possible to enforce a contractual term releasing a party from liability for gross negligence, on the grounds of public policy.^92^ English law has, however, developed differently. Within bailment law, ‘gross negligence’ came to be treated as a label or as ‘the same thing, [as negligence] with the addition of a vituperative epithet’,^93^ and it remained distinct from fraudulent or wilful misconduct.^94^ Over time, therefore, the concept has become a particularly useful tool for parties negotiating how future liabilities should be allocated between them, offering a middle way between exceptional, intention-based grounds that are onerous to prove and the broad category of ordinary negligence.

### B. Exculpatory Terms

The exclusion of a party’s liability for loss or damages absent their ‘gross negligence’, ‘wilful default’ or fraud has become a common feature of exculpatory terms in English law-governed contracts. In this context, the standard offers a useful compromise for negotiators;^95^ moreover, there is good evidence from sectors where standard terms are used,^96^ and where trade association terms inform negotiations,^97^ that this formulation has become routine, offering a valuable way of balancing risk between commercial parties.

While there is some practitioner advice that it is ‘good practice’ to define ‘gross negligence’ in service contracts,^98^ this research suggests it is not standard to do so in the commercial or banking context. However, the cost of striking a bargain based on this kind of open and undefined term can be uncertainty later on. In this respect, gross negligence is not unique; for example, Lalafaryan has explored a similar trade-off between flexibility and definition in the context of ‘Material Adverse Change’ (MAC) clauses. She shows that MAC is ‘an important yet chaotic legal concept’ which has become ubiquitous in mergers and acquisitions, and debt finance agreements, despite being ‘extremely ambiguous and uncertain’ for lenders to engage.^99^ Incorporating the ‘gross negligence’ standard offers contracting parties similar benefits at the deal stage and risks later on. However, the important point about ‘gross negligence’ in payments law is that it tracks across commercial contracts *and* market-wide regulations. Consequently, as the next section of the article will argue, there is a special importance attached to authorities about the meaning of the term.

### C. The Ardent *and Beyond*

The widely cited decision on the application of the ‘gross negligence’ standard in English civil law, the first instance decision in *The Ardent*,^100^ centred around an exculpatory term in the parties’ New York law-governed Commercial Advisory Agreement. The relevant part of the term provided that the defendants would not be liable to the claimant for ‘Damages’ arising on a range of legal grounds, except for ‘Damages resulting from acts or omissions of the Commercial Advisor [the defendant] which (a) were the result of gross negligence, (b) constituted wilful misconduct, (c) constituted fraud or (d) constituted bad faith’.^101^

Applying the clause, there were two principal issues for the court to decide. The first was determining the meaning of ‘gross negligence’ as the concept was used in this agreement, ie as ‘a saving or qualification to [this clause’s] basic principle of exception’.^102^ The second involved establishing whether the defendant’s conduct qualified as gross negligence, which was, in turn, causative of the claimant’s losses, therefore falling outside the defendant’s ‘contractual immunity’^103^ from paying damages.

Having heard from both sides’ experts, and applying New York law and public policy, Mance J held that gross negligence ‘in the present contracts’ was intended to cover conduct involving either subjective or objective recklessness ‘without any necessary implication of consciousness of the high degree of risk or the likely consequences of the conduct on the part of the person acting or omitting to act’.^104^ This conclusion was then triangulated by reference to the principles of English contractual construction,^105^ and by reference to how ‘gross negligence’ had been interpreted in English criminal law.^106^ Mance J’s conclusion on the former included the following statement, which has become the starting point for the analysis of the term across diverse English law contexts:^107^

If the matter is viewed according to purely English principles of construction, I would reach the same conclusion. ‘Gross’ negligence is clearly intended to represent something more fundamental than failure to exercise proper skill and/or care constituting negligence. But, as a matter of ordinary language and general impression, the concept of gross negligence seems to me to be capable of embracing not only conduct undertaken with actual appreciation of the risks involved, but also serious disregard of or indifference to an obvious risk.^108^

Applying English legal principles, the judge therefore reached the same conclusion about the scope of the contractual language in question as was arrived at by applying New York law and public policy. However, while the statement above has been widely cited in English civil cases and in decisions by the Financial Ombudsman Service on the UK PSR regime,^109^ the limitations of this decision need to be kept in mind. Specifically, *The Ardent* did not establish that gross negligence should be regarded as the same concept across different legal settings, nor did it definitively settle the meaning of gross negligence in English contract law. Moreover, it is ultimately a decision on the meaning of a term as intended by these contracting parties, rather than offering some kind of ‘universal’ definition.

Since *The Ardent*, there has been a steady flow of English cases interpreting ‘gross negligence’ in exculpatory clauses. Notably, there has been some divergence on the meaning of the standard, even when disputes have involved very similar drafting, as demonstrated by a line of cases arising from the actions of brokers when closing out a trading company’s positions.^110^ The exculpatory clauses in these cases sought to protect the brokers from liability on what we have seen to be standard lines, ie for any ‘losses, damages, costs or expenses’ apart from those arising from the brokers’ ‘gross negligence, wilful default or fraud’.^111^ Each of these cases focused on the allegation that the brokers were liable for ‘gross negligence’, as is usual in commercial cases involving such terms.^112^

Considering what the ‘gross negligence’ standard meant in this context, it is notable that first instance decisions came to somewhat different conclusions. In *Sucden Financial Ltd v Fluxo-Cane Overseas Ltd*, Blair J held that gross negligence could amount to the same thing as the ‘flexible’ concept of ‘simple negligence’, stating that ‘I cannot myself see that the addition of the word “gross” to negligence adds much if anything’.^113^ Similarly, in *Marex Financial Ltd v Fluxo-Cane Overseas Ltd*, David Steel J stated that it was ‘not at all clear’ what the epithet ‘gross’ added.^114^ In a different and later case also involving Marex, however, Field J considered the relevant term in more detail, concluding that there was a difference, ‘one of degree’, between negligence and gross negligence, and citing both *The Ardent* and the 2011 decision in *Camerata Property v Credit Suisse Securities (Europe) Ltd* with approval.^115^ In the latter, considering a term excluding the bank’s liability except in the cases of gross negligence, fraud or wilful default,^116^ Andrew Smith J, referencing *Armitage v Nurse*, stated that

The relevant question, however, is not whether generally gross negligence is a familiar concept in English civil law, but the meaning of the expression in these paragraphs of the Terms and Conditions. I cannot accept that the parties intended it to connote mere negligence … I accept that the distinction between gross negligence and mere negligence is one of degree and not of kind …^117^

The approach set out here by Andrew Smith J was subsequently adopted with approval in another case between sophisticated client and bank, *Winnetka*.^118^ This approach is also consistent with that adopted by the majority of the Privy Council in another 2011 case, *Spread Trustee Co Ltd*, where Sir Robin Auld (in agreement with the majority) stated that gross negligence differs from simple negligence ‘only in the degree or seriousness of the want of due care [the terms] describe. It is a difference of degree, not of kind.’^119^ Overall, therefore, these commercial cases suggest that gross negligence remains a valuable tool for parties negotiating exculpatory terms, but not a standard of conduct with a settled, distinctive meaning; as one practice note puts it, ‘Gross negligence exists but is not clearly defined’.^120^

### D. Importance for Payments Governed by Common Law

As we have seen, one of the reasons that gross negligence is important in payments law is that bank–client contracts commonly limit the liability of the bank by reference to it. For this reason, the standard was central to the recent payments case of *Federal Republic of Nigeria v JP Morgan Chase Bank NA*.^121^ Here, the state’s claim related to payments in the region of a billion dollars, for which it argued that its bank should be liable on the basis of the so-called *Quincecare* duty;^122^ meanwhile, the bank–client contract required ‘gross negligence’ for the bank to be liable, meaning the state had to show that the bank’s conduct qualified as such as part of its claim for damages.

This case demonstrates how the gross negligence standard needs to be defined, as well as applied, once it becomes relevant to the parties’ dealings. In the course of the first of these tasks, the judge described gross negligence as ‘a notoriously slippery concept’.^123^ After an extensive review of the authorities, Cockerill J emphasised the exceptional gravity of the conduct needed to breach this standard; a ‘serious lapse’ or ‘bad mistakes’ were not sufficient, rather it required ‘mistakes which have a very serious and often a shocking or startling (cf. “jaw-dropping”) quality to them’.^124^ Relatedly, this case also illustrates how contemporary payment scenarios can give rise to extensive and complex facts which make the application of the gross negligence standard challenging in practice, even once it has been defined for the purpose in hand.^125^ Ultimately, after a six-week trial, while the bank was found to have notice of a risk of fraud and failed to act, the risk was found to be so slim that this was an insufficient basis for the conduct in question to amount to gross negligence.^126^

Where do these recent decisions leave the ‘gross negligence’ standard in English civil law? First and foremost, they show that the meaning of standard depends on the legal context in which it is being used. In cases involving an exculpatory term, this means deploying the ordinary principles of contractual construction to determine what the parties intended: for example, in *Winnetka*, the judge explained that ‘since the Investment Mandate uses both the expressions “negligence” and, separately, “gross negligence”, I consider that the two cannot be intended to have the same meaning’.^127^ In *The Ardent* itself, a few paragraphs before the widely cited words set out above, the judge states that ‘the issue before me as to the scope [of the exemption clauses] is essentially one of construction, and depends on the character and working of the particular contract’.^128^

Subject to this important point, certain themes emerge from the English commercial cases. As recent authority has confirmed, ‘gross negligence’ will generally be understood as referring to conduct of a different degree, but not kind, to ordinary negligence; it extends to cases where there has been serious disregard or indifference to an obvious risk; and the standard is distinct from fraud or wilful default. On a more practical note, this section has also shown that when this standard of conduct has to be applied to real-world conduct, there can be high levels of factual complexity involved, including in cases which relate to modern payment processes.

## 4. Customer ‘Gross Negligence’ in Practice

So far, the article has explored why customer ‘gross negligence’ is pivotal to loss allocation for disputed bank payments but also how the standard can be ‘slippery’ to define and apply. This section is concerned with the practical consequences of this combination, focusing on the UK position. Specifically, it considers the adjudication of disputes which involve assessing a bank customer’s conduct against this standard, and also what these disputes tell us about the design of rules that include gross negligence as a means of delineating liability for payments.

The UK regulator has recognised the challenges for banks assessing their customers’ conduct against this standard. Following a 2015 empirical review, an FCA report ‘recognised that there are some complex concepts for firms to consider, such as … when a customer may be held liable for an unauthorised transaction due to their own gross negligence’, and that ‘this is an area that requires firms to make finely balanced judgements and implement complex legal requirements’.^129^ Furthermore, the FCA found that practices around this issue varied considerably between firms and, while there was generally good practice and ‘good efforts to reach fair judgements’ on refunds for unauthorised transactions, there were also examples of customers facing lengthy delays, burdensome paperwork and unclear terms and conditions.^130^ Tellingly, this FCA research also showed that, as regards unauthorised transactions, ‘Customers had no detailed knowledge or understanding of their rights or obligations’.^131^

Despite it being nearly 20 years since PSD1 came into force, the ‘complex concept’ of customer gross negligence under this regulation (and its successor) has not been addressed by either UK or EU courts. There have, in fact, been no UK court cases on this aspect of PSR, and few on the PSR at all. There have been ECJ decisions on the relevant PSD provisions, but these decisions have strongly upheld the ‘full harmonisation’ objective of the PSD while carefully avoiding issues, such as this one, which are reserved for national law.^132^ This is not to say, however, that disputes have not arisen about applying the term in the EU–UK regulatory context, but rather, when they have, they have not generated binding precedent. In the UK’s case, this is because it is the Financial Ombudsman Service (FOS) rather than the courts that has had to grapple with applying the ‘gross negligence’ standard and other aspects of the PSD regime in thousands of real-world payment scenarios. Accordingly, this section explores the FOS’s approach to evaluating customer conduct and explains how it is a function of both the FOS’s statutory process and the relevant legal inputs.

### A. The Financial Ombudsman Service

The FOS is an independent dispute resolution service, set up by the Financial Services and Markets Act 2000.^133^ It hears eligible complaints, including those relating to payment services, which may be brought by consumers, ‘micro-enterprises’ and smaller charities, amongst others,^134^ and is currently able to make awards of up to £445,000.^135^ In 2024/25, the service received 305,918 new complaints, up from 199,025 in the previous year, of which 35,416 were about fraud and scams (up sharply from 27,675 in 2023/24).^136^ There is evidence in the most recent FOS Annual Report that the service came under even greater pressure than it had expected during this period, as its ‘budget’ for the number of cases for 2023/24 was 210,000, and it missed all its own time targets during the year.^137^ The FOS Annual Report explains that complaints involving frauds and scams now represent over one-quarter of all banking complaints,^138^ and these cases are ‘complex, due to the evolving and ever more sophisticated tactics used by scammers’.^139^ It is also worth noting that these figures cover a period which is largely before the APP reimbursement scheme came into effect, in October 2024; this scheme may provide another significant source of work in the future.^140^ More broadly, as part of the UK government’s growth-orientated Regulation Action Plan,^141^ the performance of the FOS is currently under review^142^ and reforms have been proposed which are intended to streamline the service.^143^

The FOS’s analysis of customer conduct against the ‘gross negligence’ standard in payments regulation reflects its statutory design, as well as the legal authorities. The FOS offers a fundamentally different type of dispute resolution service to the courts, hearing complaints rather than claims.^144^ Recourse to a court in relation to a FOS final decision is by application for judicial review of the FOS’s decision making.^145^ Moreover, while the FOS’s final determinations are published on an anonymous basis (unless, in the Ombudsman’s opinion, publication would be ‘inappropriate’),^146^ they do not set precedent, as at least two firms involved in complaints relevant to this article had to be reminded.^147^

Importantly for these purposes, the underlying statutory scheme, applied and expanded upon in the FCA Handbook, provides that the Ombudsman does not take decisions in the same way as a judge, but has to ‘determine a complaint by reference to what is, in his opinion, *fair and reasonable* in all the circumstances of the case’.^148^ In doing so, the Ombudsman is required to ‘take into account relevant: laws and regulations; regulators’ rules, guidance and standards; codes of practice; and (where appropriate) what he considers to have been good industry practice at the relevant time’.^149^ The Court of Appeal has confirmed FOS decision making must be in accordance with public law principles so that it is not arbitrary or irrational, and decisions must be reasoned and take the required sources into account.^150^ However, FOS decisions do not have to strictly follow the law^151^ and, as expressly stated in the statute, an Ombudsman has powers to make an award that would not be available to the courts.^152^ The main adjudicative forum considering customer gross negligence in the payments context is therefore one empowered with a flexible and fairness-focused approach, where its decision making, rather than decisions, has to be in accordance with the law.

### B. Evaluation

The FOS website makes available ‘all the final decisions we’ve published since 1 April 2013 under the Financial Services and Markets Act 2000, as amended by the Financial Services Act 2012’; as at July 2025, selecting the ‘Banking, credit and mortgages’ category to filter these published decisions returns over 161,000 hits.^153^ There is only a limited search function provided (including searching by date range and/or by a free text search) and there are no headnotes or summaries. A total of 456 decisions are returned by a free text search for ‘“gross negligence” and “PSR”’ within the ‘Banking, credit and mortgages’ category, which span a period of nearly a decade (9 December 2015 to 6 May 2025). For the purposes of this subsection of the article, a random sample of 10% of these decisions (46 decisions) spanning this time period were reviewed and then triangulated by further text-based searches of the same database where a theme required further investigation. The aim of this review was to provide insights into the nature and application of the customer gross negligence standard in practice, which could be usefully woven into other findings in the article.

#### (i) The nature of FOS payments disputes

FOS decisions vividly illustrate how modern payment processes sit at the intersection of technology, finance and social life. As a result, a single complaint can involve complex, wide-ranging and, for the customer, deeply personal events, while there can be numerous intermediaries between payer and payee. These factors intensify the challenge when it comes to applying the UK PSR.

By way of example, FOS adjudicators have had to assess the question of a customer’s gross negligence in the context of the following: a scam involving phone calls to the victim who was tricked into sharing one-time passcodes (OTPs) from his bank, which were then used to authenticate purchases the victim said he did not make;^154^ a complaint involving a stolen phone, which gave rise to complex issues of fact around the interaction between the phone, the victim’s bank account, ApplePay and the security features of each;^155^ a phone call from fraudsters to a company director who was tricked into completing certain steps on his banking app, sharing OTPs and scanning a QR code, which gave fraudsters access to the company’s account;^156^ and a substantial fraud involving an investment scam publicised on social media, whereby the victim was persuaded to use remote access software to set up a cryptocurrency wallet and to share OTPs which enabled payments from his account and credit card.^157^ Relatedly, published FOS decisions regularly state that they are able to record only a selective account of the facts, and/or note the ‘incomplete, inconclusive or contradictory’ evidence^158^ produced by the parties. The complexity inherent to complaints about payments therefore makes for a challenging setting in which to evaluate customer conduct, in particular given that the FOS is required to take into account ‘all the circumstances of the case’.

#### (ii) Approach to ‘gross negligence’

In 26 of the 46 decisions reviewed for this purpose, a bank customer’s conduct was evaluated in terms of ‘gross negligence’ and a conclusion reached on this point (in the other cases, the issue was mentioned but fell away before an evaluation was required). In four of these decisions, the customer was found to have acted in a way that constituted gross negligence and, as a result, they were denied an award in respect of a disputed payment.

It is notable that the legal starting point for the evaluation of customer conduct against the gross negligence standard varies across these FOS decisions. Some decisions do not engage with the legal background to the standard at all, but briefly state either that it means ‘very significant carelessness’^159^ or that there is a high bar for gross negligence,^160^ while some decisions simply record that conduct of this kind has not been established, without explaining what this exercise involved.^161^ By contrast, other FOS decisions take time to set out (various) legal frameworks, citing a range of sources. One decision from 2023 expressly states that there is ‘no definitive definition of gross negligence’ in the context of the UK PSR, before setting out an understanding of the term that echoes the well-known passage in *The Ardent* with its two elements, ie that it requires ‘a very significant degree of carelessness and a serious disregard to an obvious risk’.^162^ This approach is seen in at least one other FOS decision in this sample.^163^ Two different 2019 decisions took the hunt for the meaning of the gross negligence standard further by citing *The Ardent* at some length, alongside Recital 72 of the PSD2 and the FCA Guidance on the latter. The last two of these sources were discussed in both decisions even though they came into force *after* the relevant events in those complaints, to which PSD1 applied. As both these 2019 decisions put it, using the same words:

Although neither of these [sources] is directly relevant to this case, they are of value as a relevant consideration in the absence of contemporaneous interpretative guidance, and because they inform the meaning of a concept that has been in place for some time (in the Banking Code).^164^

It is striking that these decisions emphasise the continuity from the Banking Code to the PSR, as highlighted earlier in the article, as well as demonstrating creativity in terms of the sources which they are taking into account in order to attribute meaning to this standard. The well-known quote from *The Ardent*, meanwhile, is reproduced, or the decision referred to, in over two dozen FOS decisions in the ‘Banking, credit and mortgages’ category overall.

Rather than reference legal authorities, other FOS decisions assess gross negligence by setting up a direct comparison between the customer involved in the complaint and the ‘reasonable person’, for example:

We would consider gross negligence to be a lack of care that goes significantly beyond what we would expect from a reasonable person. So, the consumer’s actions must be assessed against what a reasonable person in Mr D’s position would have done, and their actions should only amount to gross negligence if what they did fell far below the standard expected of a reasonable person.^165^

This comparative approach to assessing the conduct of the customer, in turn, reveals the Ombudsman making significant assumptions about the vulnerability of the ‘reasonable’ customer in the modern banking system, in particular in the context of scams. Moreover, assumptions about the vulnerability of the reasonable customer were found across a wide range of published decisions; they were not confined to customers who might traditionally have been regarded as vulnerable, and were found in decisions involving both authorised and unauthorised payments. For example, one company director who was manipulated by fraudsters into forwarding an email and sharing OTPs was found not to have acted with gross negligence because ‘After all, Mr G, like most people, isn’t an expert in fraud or Cashplus’s procedures. So I can see how the fraudster’s instructions didn’t ring alarm bells.’^166^ Similarly, it was found not to be gross negligence when a victim was tricked as part of a ‘clever and convincing scam’ involving emails and sharing of passcodes,^167^ nor when a victim received a call purportedly from his bank and shared his full card details and PIN. In this last decision, it was found to be relevant that:

As Mr A was then satisfied he was speaking to his bank, I can’t say it was then unreasonable he complied with its request to provide certain other personal information, including his PIN. I think many other people would—and we know have from the cases we see—followed the caller’s instructions and complied with what they asked for.^168^

These FOS decisions, alongside others on the same lines,^169^ therefore seem to reflect an assumption that the ‘reasonable person’ has few defences when faced with ‘convincing’ and/or tech-based ‘social engineering’ scams. As a result, conduct such as openly sharing a PIN is treated as a symptom of a scam rather than as action which could qualify as gross negligence, which we can compare with findings of gross negligence in complaints involving unauthorised transactions rather than APP scams, when customers had stored PIN details with stolen cards and in an ‘easily detectable form’.^170^ This sample did include instances where the FOS found victims of scams to have acted with gross negligence,^171^ but it is clear that the sinister phenomenon of ‘social engineering’ opens up important and distinctive questions around customer vulnerability and how to define expectations of customers. Certain fact patterns involving social engineering also call into question the logic of maintaining distinct legal regimes for authorised and unauthorised payments.

As we have seen, the FOS is empowered to adopt a more flexible approach than the courts and to reach decisions which are ‘fair and reasonable in all the circumstances of the case’. It is arguable, therefore, that while there are challenges around defining the gross negligence standard from the UK PSR and applying it to real-world scenarios, the FOS is well placed to do so. However, the flexibility of the FOS’s process and outcomes does not necessarily mean that the rules that it is required to take account of could not be improved. Furthermore, the same underlying rules apply to other stakeholders, including account-holding banks, which are not provided with the same flexibility when it comes to implementing regulation. The final part of the article therefore draws together reasons why the design of the ‘gross negligence’ limitation on bank liability should be rethought and considers what form a change might take.

## 5. Implications

Building on the article’s findings thus far, this section proposes that, in keeping with other EU-originated UK legislation in which this private law standard is embedded, there should be an express definition of gross negligence ‘purpose-built’ for payments regulation. The form and substance of that definition are explored below. Because the latter opens up questions around regulatory rationale, this issue is also addressed, drawing upon the earlier analysis of the groundbreaking Banking Code. While this section primarily focuses on UK regulation, following on from the preceding discussion of English common law and the work of the FOS, it also draws on a parallel debate ongoing in relation to the EU’s proposed reforms to PSD2.^172^ Specifically, this section considers a recent recommendation by van Praag and participating authors that gross negligence should be expressly defined for the purposes of the Proposed EU Payment Regulation.^173^ Overall, the purpose of this section is not to present an express definition itself, because the job of drafting an express definition requires a broader and different analysis to that conducted in this article (for example, extending to a wider analysis of evidence from decision making at firm and FOS level). Rather, the aims of this section are to explain how the article’s analysis of the gross negligence standard across payments law leads to the conclusion that an express definition would improve the status quo, and to explore how it should inform the substance of such a definition.

### A. Express Definition: Form

There are several reasons why the analysis so far supports the concept of ‘gross negligence’ being expressly defined for the purposes of UK payments regulation. First, it suggests that the status quo is no longer appropriate, given the transformation which has taken place in the payments sector over the last 30 years. More than three decades since the standard was adopted in the first Banking Code, contracting parties continue to deploy (and occasionally litigate) this undefined term on a routine basis, but this is no longer an appropriate approach for the vast numbers of extensively regulated and increasingly diverse payment relationships falling under the EU–UK regime and the UK’s new APP reimbursement scheme. As the objectives of fairness and transparency in the bank–customer contract were amongst those which drove the introduction of the Banking Code in 1992, the same objectives require a new approach today. Consequently, the EU-derived rules considered in this article should follow the approach already adopted in other Regulations and expressly provide what the gross negligence standard means in this context.

Following on from this point, we have seen how the burden of defining and applying this aspect of EU–UK payments regulation has become more complex and demanding due to ongoing social and technological trends, as highlighted by FOS decisions. This complexity can be intense in relation to payment-related frauds, which are becoming endemic. Given the sheer value and volume of modern payments, any such burden quickly scales up. As noted, there are mounting pressures on FOS processes, which suggests that implementing a consistent definition of this standard would be operationally valuable. Moreover, an express definition promises efficiencies for other stakeholders applying these standards in practice, including payment service providers, thereby benefiting the customers involved and potentially helping to bolster confidence in the banking system.

Relatedly, an express definition also makes sense in terms of certainty and transparency. The task of defining and applying the customer gross negligence standard currently falls to banks in the first instance, around which process there is little transparency at present, and therefore little possibility of monitoring approach or outcomes in depth across the sector. At the FOS level, this article has shown that the Ombudsman’s analysis of customer conduct against this standard does not, at present, have a standard starting point. Moreover, neither banks’ nor the FOS’s very extensive decision making on this issue is currently used as it could be to inform awareness-raising efforts. A definition could therefore provide a basis both to develop monitoring and to inform stakeholder education; it could also increase consumer confidence in the banking system, including by drawing attention to the relatively limited grounds on which otherwise payable refunds for disputed payments may be denied.

Finally, the findings above suggest that there is an appetite for an express definition. FOS decisions cite FCA guidance, even where complaints involve facts pre-dating it, demonstrating the Ombudsman’s hunt for authority on this point. At present, however, even this particular guidance is limited, falling short of a definition; it is also circular with respect to the Recital in PSD2, which reserves this matter for national law. The use of certain widely cited passages from *The Ardent*, referenced in over two dozen FOS decisions, adds weight to the case for an express definition. *The Ardent*, as well as more recent decisions on exculpatory terms found in commercial contracts between sophisticated parties, is ultimately an exercise in contractual interpretation aimed at determining the intentions of the parties in question. There is a different goal in this statutory setting and, as a result, the analysis should be more focused from the outset on the purpose of this loss allocation regime.

In the course of dismissing a claim for judicial review of a FOS decision, Rix LJ suggested that the overall purpose of the FOS scheme was to balance the provision of ‘an efficient and cost-effective and relatively informal type of alternative dispute resolution’, on the one hand, with upholding ‘transparency, consistency and accessibility’ as to the principles and determinations of the Ombudsman, on the other.^174^ In the context of UK payments regulation, an express definition of gross negligence could provide valuable support for the FOS as it performs this difficult balancing act, while also improving the approach of other stakeholders. Of course, it is also relevant to note that the FOS decides what is ‘fair and reasonable’ in each case, rather than what is strictly required by the law. Ultimately, this remit softens the risk of an express definition being applied mechanically to unfair ends.

### B. Express Definition: EU Insights

Debates around the EU’s proposed reforms to payments legislation have included discussion of the meaning of a customer’s ‘gross negligence’, because it appears as a limit on a new reimbursement right for a certain, narrow type of APP fraud. As van Praag and participating authors put it in a recent European Banking Institute (EBI) working paper on the EU’s proposed rules for APP fraud, various EU authorities have considered the ‘difficult question’ about ‘to what extent the concept of gross negligence should be harmonised in the PSR’ and have made different suggestions about how to achieve this outcome.^175^

EU analysis cannot, of course, simply be transposed into UK-centric debates.^176^ One particular limitation in this context is that the EU is pursuing specific objectives through the reform of payments law, the principal of which is the greater harmonisation of the legal framework across Member States. For example, the European Parliament’s suggested new Recital for the proposed PSD3 states:

(82a) Taking into account the fact that the term ‘gross negligence’ is interpreted in very different ways across the Union the [European Banking Authority] should issue guidelines on how that concept is to be interpreted for the purpose of this Regulation.^177^

Though EU analysis has its limits in the UK context, it still offers useful insights. Most significantly for this section, the majority of authors of the recent EBI working paper also recommend an express definition of ‘gross negligence’. In terms of what such a definition should look like, the EBI working paper rejects the idea of a list of ‘arbitrary’ and ‘non-exhaustive’ criteria for ‘gross negligence’ by customers, on the grounds that this approach risks obsolescence and overbroad interpretation by banks.^178^ Instead, it recommends that a definition be included in the new EU Payment Regulation, suggesting it could follow the approach used in Dutch case law, supplemented with ‘concrete examples’ in the Recitals.^179^ The EBI working paper makes the further point that an express definition would also facilitate regulators’ supervision of banks, and would enable the regulator to act where a regulated entity ‘too often refuses to compensate based on gross negligence’.^180^ While these recommendations reflect the specific aim of harmonisation across Member States, they nonetheless also offer valuable insights for the UK.

### C. Express Definition: Substance

This article’s findings suggest that an express definition of gross negligence for the payments context should be shaped by several factors, which are considered later in this section. First and foremost, however, the overall design of this definition needs to be informed by clear and prioritised policy goals. As this article has shown, loss allocation between banks and customers has become a powerful regulatory tool, with much at stake in terms of design. Indeed, the particular importance of policy for the design of payments regulation has been acknowledged by courts as well as by other commentators. In *Philipp*, the Supreme Court emphasised the role of regulators in designing reimbursement rules for APP fraud ‘which may not have a principled basis but are considered to promote the common good by achieving an appropriate trade-off or compromise between different policy goals’.^181^ Similarly, the EBI paper flags up that the exact design of any EU regime which ultimately spreads the costs of APP fraud around bank customers as a group will ‘in the end [be] deeply political and depends on the type of society we want to build’.^182^

At this point, it is useful to remind ourselves how and why the gross negligence standard came to feature in UK payments regulation, ie in the groundbreaking 1992 Code of Banking Practice, designed to promote fairness, transparency and customers’ trust in the banking system.^183^ While diverse objectives continue to accrete around the modern payments sector, revisiting the original purpose behind this regulatory design helps to refocus the debate. Specifically, the analysis developed in the article thus far suggests the following factors should be taken into account in drafting an express definition of gross negligence standard designed to promote these objectives, which should apply across UK payments regulation:

- The FOS decisions analysed above provide numerous examples where a single scenario can give rise to both authorised and unauthorised payments, and where this categorisation is arguable on the facts.^184^ The distinction between the two types of losses in certain ‘social engineering’ scam scenarios can become especially blurred. Consequently, a single definition for ‘gross negligence’ spanning both legal regimes would be both functional and fair, in that it would help to treat like cases alike. An expressly defined conduct standard applying to both types of payments would also enable clearer and more transparent guidance to emerge and would facilitate both awareness raising and the supervision of parties applying these standards.
- The UK mandatory reimbursement scheme for APP fraud currently weaves gross negligence into a wide-ranging list of specific requirements which go beyond the payment process, including around reporting the scam to relevant authorities. This approach is overcomplex and potentially undermines important fraud-prevention measures included in this definition. It would therefore be clearer and more effective in terms of upholding the different aims of this scheme if stand-alone requirements such as fraud reporting, on the one hand, and conduct standards associated with the payment process, on the other, were addressed separately.
- The exercise of drafting an express definition of this kind also provides an opportunity to explore and update the regulatory approach to bank customers’ vulnerability. This is an issue on which fairness to customers requires a more inclusive, transparent and detailed approach than in the past. In particular, the article has highlighted how the question of how best to understand vulnerability when evaluating conduct for gross negligence has become more challenging in the context of technology-enabled banking and social engineering, creating some inconsistent outcomes. Interestingly, the EBI paper also considers the interaction of vulnerability and customer conduct standards, suggesting that that vulnerability should not ‘play a key role when evaluating gross negligence on behalf of the [payment services user]’, partly because ‘the mere fact that the PSU became a victim of fraud, shows he is vulnerable for this type of fraud. Also, it would reinforce stereotypes of who is vulnerable.’^185^ As indicated, the current position in the UK seems to have some important differences with this approach, but a consistent and principled overall approach seems lacking at present. In sum, it would be appropriate for liability standards in loss allocation rules to be informed by an updated and more detailed understanding of a reasonable customer’s vulnerability at key points in the modern payment process, providing a more sophisticated framework for adjudicators to apply to conduct on a case-by-case basis.
- Relatedly, this article suggests that there is value in developing more evidence-based guidance for all stakeholders alongside an express definition of ‘gross negligence’, for example, around the nature and impact of personalised security warnings received during the payments process. Such guidance should be informed and periodically updated by data gathered from the FOS and banks (FOS decisions sometimes state that they take into account customer conduct they see on a recurring basis when assessing ‘gross negligence’; it would be helpful if these insights were made available in a more accessible way). More specific *ex ante* guidance would also be helpful when it comes to promoting transparency, awareness raising and customer education. On the other hand, the risk of introducing more precise guidance of this kind is an arbitrary or unfair outcome in a particular case. However, this is a matter of careful drafting (perhaps adopting a ‘case study’ approach) while, as a backstop, it is important to remember that the FOS is not bound to apply the law in the same way as a court, and it ultimately reaches decisions on the basis of what is ‘fair and reasonable’ in each case.

## 6. Conclusion

This article began by explaining why payments law matters. As the subsequent discussion has shown, the scale and stakes in this sector are vast, to the extent that the meaning of a specific legal concept which is embedded at critical junctures across payments law has far-reaching consequences. In this context, the article has examined the meaning and significance of the standard of gross negligence across different areas of payments law, and it has ultimately drawn attention to the shortcomings of the status quo. Specifically, the article has explored how the English common law concept of ‘gross negligence’ is routinely deployed in contracts to delineate the parties’ liabilities, offering a useful ‘agree now; define and apply later’ trade-off. Having been interpreted and applied by the courts on numerous occasions, the standard remains ‘slippery’; nonetheless, the article has addressed why and how it has been folded into vitally important regulatory regimes which apply to both authorised and unauthorised payments, amounting to billions of transactions each year.

Based on insights from common law and from FOS decisions, however, the article has argued that this standard, in its undefined form, is no longer an appropriate way of determining liability in respect of the vast numbers of diverse payments which fall within the scope of payments regulation. In the regulatory context, the openness around the meaning of the gross negligence standard for customer conduct generates a burden at the application stage, most obviously falling on the FOS. It also compromises transparency, certainty and awareness raising, including around the growing number of complex scenarios involving sophisticated frauds.

Ultimately, this article has suggested introducing a purpose-built, express definition of the gross negligence standard for UK payments regulation following the example, but not the wording, of other EU-derived regulations. It has argued that drafting a suitable definition should be informed by the original regulatory context and that a definition should take specific factors into account in order to promote fairness, transparency and trust in the payments system. These factors included the nature of vulnerability in an era of tech-fuelled scams, and should be developed further in light of a broader review of FOS decisions and stakeholder analysis than that undertaken in this article. Engaging with the meaning and application of ‘gross negligence’ therefore not only enables us to track significant legal issues right across the payments sector, but also suggests how we might improve a regime upon which millions of stakeholders rely.

## Footnotes

1 UK Finance Ltd, ‘UK Payment Markets Summary’ (July 2024) 17. Remote banking is defined as ‘online banking via a computer, mobile banking via an app on a smartphone or tablet, or [the customer] calling their bank via a telephone banking service’ ibid 13.

2 ibid 15.

3
*Federal Republic of Nigeria v JP Morgan Chase Bank NA* [2022] EWHC 1447 (Comm) [325] (Cockerill J).

4 Unless otherwise indicated, the article uses the term ‘bank’ to cover the various types of entities providing payment services (including banks, building societies and electronic money institutions). When a specific legislative definition is relevant to the analysis, that term is used; see eg s 2B. The term ‘bank’ is used otherwise for readability and because the distinctions between various types of service providers in the payment sector are not an issue of relevance for this article.

5 Discussed in ss 2B and 2C below.

6 s 2D.

7 UK Finance Ltd, ‘Annual Fraud Report 2025’, 12.

8 ss 2 and 5 address ongoing reviews of relevant payments regulations.

9
*Foley v Hill* [1848] II House of Lords Cases (Clark’s) 28.

10
*Philipp v Barclays Bank UK Plc* [2023] UKSC 25 [16].

11 UK Finance Ltd, ‘Annual Fraud Report’ (n 7) 46 states that fraud involving international payments was up 93% by value between 2023 and 2024; it also suggests that the UK’s new reimbursement scheme (s 2D below), which also does not cover international payments, may be driving this trend, ibid 5.

12
*Philipp* (n 10) [29]–[30].

13 ibid [97]–[100].

14 For a comparative analysis, see Sandra Booysen, ‘Consumer Protection and the Court’s Role in Shaping the Bank–Customer Contract’ (2019) 135 LQR 437.

15
*London Joint Stock Bank v Macmillan* [1918] AC 777.

16
*Greenwood v Martins Bank* [1933] AC 51.

17
*Tai Hing Cotton Mill Ltd v Liu Chong Hing Bank Ltd* [1986] AC 80.

18 ibid 103: ‘it is always open to a banker to refuse to do business save upon express terms including such a duty’ (Lord Scarman).

19
*Federal Republic of Nigeria* (n 3), discussed further in s 3D.

20 British Bankers’ Association, ‘Good Banking: Code of practice to be observed by banks, building societies and card issuers when dealing with personal customers’ (December 1991) (Banking Code; copy on file with the author).

21
*Banking Services: Law and Practice, Report by the Review Committee Chaired by Professor R.B. Jack* (Cm 622, 1989) ch 16 (*Jack Cttee Report*). An overview of this report and measures which followed is set out in Anu Arora, ‘Banking law review since the Jack Committee report on banking services’ (1991) 12(9) Co Law 182.

22
*Jack Cttee Report* (n 21) [2.13].

23 ibid [2.07].

24 ibid [2.09]–[2.10].

25 ibid [17.05].

26 ibid ch 2 considers the ‘Changing Scene’ for banks and customers at the time and the fitness of existing law.

27 ibid [17.03] and [17.10].

28 ibid [17.08].

29 Banking Code (n 20).

30 ‘Draft Code of Banking Practice’ Practical Law UK Legal Update 7-100-5630 (1 April 1991).

31 ibid, referencing para 21.4 of the draft Code (emphasis added).

32 ‘Code of Banking Practice’ Practical Law UK Legal Update 4-100-4515 (1 January 1992).

33 ibid; Banking Code (n 20) paras 18.4 and 18.5.

34 Commission Recommendation of 17 November 1988 concerning payment systems and in particular the relationship between card holder and card issuer 88/590/EEC [1988] OJ L317/55, s 8; addressed in *Jack Cttee Report* (n 21) ch 3 and set out in Appendix P.

35 Directive (EU) 2007/64/EC of 13 November 2007 on payment services in the internal market, amending Directives 97/7/EC, 2002/65/ EC, 2005/60/EC and 2006/48/EC and repealing Directive 97/5/EC [2007] OJ L319/1 (PSD1).

36 Directive (EU) 2015/2366 of 25 November 2015 on payment services in the internal market, amending Directives 2002/65/EC, 2009/110/EC and 2013/36/EU and Regulation (EU) No 1093/2010, and repealing Directive 2007/64/EC [2015] OJ L337/35 (PSD2).

37 European Commission, ‘Payment Services: Revised Rules to Improve Consumer Protection and Competition in Electronic Payments’ (28 June 2023) QANDA/23/3544; discussed further in s 5B.

38 Payment Services Regulations 2009, SI 2009/209.

39 Payment Services Regulations 2017, SI 2017/752 (UK PSR).

40 Under the European Union (Withdrawal) Act 2018, s 2.

41 eg PSD2 introduced rules covering new payment services and intermediaries including ‘payment initiation service providers’, and addressed ‘strong customer authentication’.

42 Unless otherwise indicated, reference in the article to the ‘PSD regime’ and ‘EU regime’ is to PSD2.

43 Excluded payments are listed in PSD2, art 3, including those made by cash or paper cheques, and transactions within a securities settlement system or between clearing houses and central banks.

44 PSD2, art 2. The equivalent, though now slightly broader, provisions in the UK rules are found in UK PSR, reg 63.

45 PSD2, art 4(11) and Title IV (‘Rights and Obligations in Relation to the Provision of Payment Services’). Electronic money is included in the scope of Title IV, subject to potential derogations by national regulators, PSD2, art 63(3).

46 PSD2, art 61(1) provides that payment service users who are not consumers may contract out of specified provisions of Title IV, including those relating to reimbursement for unauthorised payments. Under the UK PSR, parties must not be ‘a consumer, a micro-enterprise or a charity’ to contract out. UK PSR, reg 63(5).

47 eg Case C-337/20 *DM, LR v Caisse Régionale de Crédit Agricole Mutuel (CRCAM)—Alpes-Provence* [2021] ECLI:EU:C:2021:671.

48 PSD2, art 64 states that a transaction is considered authorised if it is one to which the payer gives their consent in the form agreed between the payer and their payment service provider.

49 PSD2, art 73(1).

50 Including the rule that the bank has the burden of proof to establish that a transaction was authorised if it is flagged as unauthorised by the customer, PSD2, art 72(1).

51 PSD2, art 71(1).

52 By way of illustration, ‘Barclays, Terms and Conditions for Personal Customers’ (June 2025) 23 <www.barclays.co.uk/important-information/personal-terms-conditions/> all links accessed 18 August 2025.

53 PSD2, art 74(1), with an exclusion from this qualification where the loss, theft or misappropriation was not detectable by the payer prior to payment unless the payer was acting fraudulently or where the loss is caused by acts or omissions on the payment service provider’s part.

54 ibid.

55 PSD2, art 4(14): ‘a personalised device(s) and/or set of procedures agreed between the payment service user and the payment service provider and used in order to initiate a payment order’. Examples include a bank card and banking app.

56 PSD2, art 4(31): ‘personalised features provided by the payment service provider to a payment service user for the purposes of authentication’. Examples include a PIN or one-time passcode (OTP) sent to a mobile phone.

57 PSD2, art 69.

58 PSD1, Recital 33; PSD2, Recital 72.

59 ibid.

60 Regulation (EC) No 1060/2009 of 16 September 2009 on credit ratings agencies [2009] OJ L302/1, art 35a(1), inserted by Regulation (EU) No 462/2013 of 21 May 2013 amending Regulation (EC) No 1060/2009 on credit rating agencies [2013] OJ L146/1, art 1(22).

61 ibid; PSD2, Recital 35.

62 Credit Ratings Agencies (Civil Liability) Regulations (2013) SI 2013/1637, reg 4.

63 Financial Conduct Authority, ‘Payment Services and Electronic Money−Our Approach’ (v 6, November 2024) paras 8.221 and 8.222 (FCA Guidance).

64 An overview of this type of fraud is set out in Payment Systems Regulator (PSR), ‘Fighting Authorised Push Payment Fraud: A New Reimbursement Requirement: Response to September 2022 Consultation (CP22/4)’ (June 2023) PS23/3.

65
*Philipp* (n 10).

66 Bank of England, Annex A to the CHAPS Reference Manual—CHAPS Reimbursement Rules <www.bankofengland.co.uk/-/media/boe/files/payments/chaps/chaps-reimbursement--rules-annex-1.pdf>.

67 Pay.UK, ‘FPS Reimbursement Rules’ (v 3.5, 4 December 2024) (APP reimbursement scheme). FPS is typically used for consumer payments, CHAPS for corporate and high-value payments. The average CHAPs payment for 2023–24 was just over £1.5million. Pay.UK, ‘Quarterly Statistical Report for Q4 2024’. Given its greater relevance for consumer payments and by volume of annual payments, the focus here is the set of rules applying to FPS payments, though the relevant terms of the two schemes are nearly identical.

68 Jo Braithwaite, ‘“Authorized Push Payment” Bank Fraud: What Does an Effective Regulatory Response Look Like?’ (2024) 10(2) Journal of Financial Regulation 174. Note that the scheme covers ‘Consumers’, defined as individuals, microenterprises and charities. APP reimbursement scheme (n 67) s 10.4.

69 ibid s 3.11.

70 ibid s 3.6, setting out the standard as provided by Payment Systems Regulator, ‘Specific Requirement 1: The Consumer Standard of Caution Exception’ (December 2023).

71 The regulator’s guidance suggests this might be the case where the victim is fearful because of a ‘particularly sophisticated scam’: PSR, ‘Guidance: Authorised Push Payment Fraud Reimbursement: The Consumer Standard of Caution Exception Guidance’ (December 2023) paras 1.31 and 1.32.

72 ibid para 3.6.

73 APP reimbursement scheme (n 67) s 10.4, citing FCA, ‘Guidance for Firms on the Fair Treatment of Vulnerable Customers’ (updated 7 March 2025) <www.fca.org.uk/publications/finalised-guidance/guidance-firms-fair-treatment-vulnerable-customers>.

74 Lending Standards Board, ‘Contingent Reimbursement Model Code for Authorised Push Payment Scams’ (v 4.0, 17 October 2023) R2(3)(a).

75 PSR, ‘Guidance’ (n 71). The following observations draw on paras 1.4-1.7 of the Guidance.

76 ibid para 1.7.

77 PSR, ‘Fighting Authorised Push Payment Fraud’ (n 64) para 1.20, adding ‘With better data and a richer understanding of fraud, we will continue to evolve this policy framework to drive APP fraud out of UK payments’.

78 Commission, ‘Report from the Commission on the review of Directive 2015/2366/EU of the European Parliament and of the Council on payment services in the internal market’ COM (2023) 365 final, 10–11.

79 Commission, ‘Proposal for a Regulation on payment services in the internal market and amending Regulation (EU) No 1093/2010’ COM (2023) 367, art 59 and Recital 80 (Proposed EU Payment Regulation). Discussed further in s 5 below.

80 Opinion of the European Central Bank of 30 April 2024 on a proposed Regulation and Directive on payment and electronic money services (CON/2024/13) (C/2024/3869) OJ C, 19.6.2024, 3.1.3.

81 Opinion of the European Economic and Social Committee on […] c) Proposal for a Regulation on payment services in the internal market and amending Regulation (EU) No 1093/2010 (COM (2023) 367 final—2023/0210 (COD)) (5 March 2024) (C/2024/1594) 3.2.6.

82 Emanuel van Praag, ‘Authorised Push Payment Fraud: Suggestions for the Draft Payment Services Regulation’ (2 May 2025) EBI Working Paper Series No 190, 3, conclusion (iv). A list of authors participating in this paper is provided at ibid, fn 1 (EBI paper) which states as follows: ‘The coordinating author of this article is E.J. van Praag (the Netherlands). Authors from the following countries in alphabetical order of countries participated in the drafting of this article: Belgium (R. Steennot), Norway (M. Eidsand Kjørven, V. Wold, L.P. Halvorsen Omland), Portugal (M.R. Guimarães), The Netherlands (K.J.O. Jansen, G.S. Breukelaar), United Kingdom (A. Fejõs). All authors participated in the discussions about the entire article, but may not necessarily agree with everything written here.’

83 ibid s 6. The EBI paper’s recommendations around the gross negligence standard are considered in s 5 below.

84 The absence of a regulatory definition was noted in *Winnetka Trading Corporation v Julius Baer International Ltd* [2011] EWHC 2030 (Ch) [16].

85 Nolan has argued that reintroducing gross negligence in tort could be a useful way to vary the standard of care in favour of the defendant: Donal Nolan, ‘Varying the Standard of Care in Negligence’ (2013) 72(3) CLJ 651, 674–6.

86 This offence has been subject to strong criticism on the grounds that the gross negligence standard is ‘a slippery concept for juries’, which should therefore ‘be abandoned’: Anne Lodge, ‘Gross negligence manslaughter on the cusp: the unprincipled privileging of harm over culpability’ (2017) 81(2) JCL 125, 130.

87 eg considered in Christopher Arnull, ‘A sterile and semantic debate about negligence’ (2020) 36(1) PN 19, 32.

88
*Armitage v Nurse* acknowledges both positions, ie the court held that parties could, as a matter of law, agree to exclude trustees’ liability for gross negligence while it also acknowledged the widely held view that they ‘should not be able to’: *Armitage v Nurse* [1998] Ch 241, 256 (Millett LJ, with whom Hirst and Hutchison LJJ agreed).

89 David Kershaw, *The Foundations of Anglo-American Corporate Fiduciary Law* (CUP 2018) 158.

90 ibid 239.

91
*Colnaghi, U.S.A. Ltd et al v Jewelers Protection Services Ltd* 81 NY 2d 821 (1993) (Court of Appeals of New York), 823. For a practitioner discussion of the issue, see David Shine, ‘Contractual Applications of Negligence/Gross Negligence Standards: Considerations Under New York Law’ (2005) 8(10) The M & A Lawyer 10, 12.

92
*Kalisch-Jarcho Inc v City of New York* 58 N.Y. 2d 377 (1983) [6]–[8], quoted and discussed in *Red Sea Tankers Ltd v Papachristidis (The Ardent)* [1997] 2 Lloyd’s Law Reports 547, 581–2.

93 The phrase is from *Wilson v Brett* (1843) 11 M&W 111, 116 (Rolfe B), quoted and discussed in Kershaw (n 89) 160–1. In Millett LJ’s judgment in *Armitage* (n 88) 254, the reference for the same phrase is Willes J in *Grill v General Iron Screw Collier Co* (1866) L.R. 1 C.P. 600, 612. The evolution of gross negligence in English bailment law is discussed in-depth in Kershaw (n 89) 160–8.

94
*Spread Trustee Co Ltd v Hutcheson* [2011] UKPC 13, [51] (Lord Clarke of Stone-cum-Ebony JSC, with whom the majority agreed).

95 As addressed in Arnull (n 87); Shine (n 91) 10. Guidance for practitioners negotiating limitation clauses generally also addresses the possibility of including negligence or gross negligence, eg Practical Law Commercial, ‘Limiting Liability: What Is “Gross Negligence”?’ Practical Law UK Practice Note w-026-1684, accessed 8 December 2025.

96 Such as the brokers’ terms considered later in this section, or the standard trustee exemption clauses considered in cases including *Spread Trustee* (n 94) [88]–[89], [103]–[107].

97 eg Association of Corporate Treasurers, *A Borrower’s Guide to the LMA’s Investment Grade Agreements* (6th edn, Slaughter & May 2022) 312, on terms addressing the potential liability of Agents.

98 Arnull (n 87) 31–2, concluding with a suggested definition for contracting parties.

99 Narine Lalafaryan, ‘Material Adverse Change Uncertainty: Costing a Fortune if not Corporate Lives’ (2021) 21(1) JCLS 39, 39 and 40.

100
*The Ardent* (n 92).

101 The relevant clause of the Commercial Advisory Agreement is set out in full in ibid 579.

102 ibid 580.

103 ibid 588.

104 ibid 586.

105 ibid 586.

106 Discussing *R v Adamoko* [1995] 1 AC 171, ibid 586–7.

107 See later in this section, and ss 3D and 4B(ii) below.

108
*The Ardent* (n 92) 586.

109 See eg this part of Mance J’s decision reproduced and applied in FOS decisions DRN6654071, DRN2339957 and DRN6311337, discussed further in s 4B below.

110 A fact pattern typical of these broker–client cases is set out at *ED&F Man Commodity Advisers Ltd & Ors v Fluxo-Cane Overseas Ltd, S/A Fluxo-Comercio E Assessoria Internacional* [2010] EWHC 212 (Comm) [1]–[10].

111 eg *Marex Financial Ltd v Fluxo-Cane Overseas Ltd & Anor* [2010] EWHC 2690 (Comm) [14], citing the parties’ ‘Terms of Business’, clause 15.1 ‘General exclusion’.

112 As Sir Robin Auld put it in his concurring opinion in *Spread Trustee* (n 94) [117]: ‘Gross negligence, like negligence not so qualified, may be committed in good faith and, therefore, without dishonesty or wilfulness. Indeed, dishonesty-an inherent ingredient of fraudulent or wilful misconduct-is the antithesis of negligence, an inadvertent falling short of a duty to take reasonable care in all the circumstances.’ The same point is made in *Springwell Navigation Corp v JP Morgan Chase Bank & Ors* [2010] EWCA Civ 1221 [33], [94].

113
*Sucden Financial Ltd v Fluxo-Cane Overseas Ltd* [2010] EWHC 2133 (Comm) [54] (Blair J). However, given that the claimant could not establish ‘mere’ negligence on the part of the broker, there was no need for the court to consider this point further: ibid [65].

114
*Marex* (n 111) [93] and [106] (David Steel J).

115
*Marex Financial Ltd v Creative Finance Ltd & Anor* [2013] EWHC 2155 (Comm) [67] (Field J).

116
*Camerata Property Inc v Credit Suisse Securities (Europe) Ltd* [2011] EWHC 479 (Comm) [3] (Andrew Smith J).

117 ibid [161], referencing *Armitage* (n 88) 254C–D.

118
*Winnetka* (n 84) [16].

119
*Spread Trustee* (n 94) [117], also referring to *Armitage* (n 88).

120 Practical Law (n 95).

121
*Federal Republic of Nigeria* (n 3).

122
*Barclays Bank v Quincecare Ltd* [1992] 4 All ER 363, establishing a bank’s duty of care when payment instructions come from an agent of the customer. Discussed further in Braithwaite (n 68).

123
*Federal Republic of Nigeria* (n 3) [325] (Cockerill J).

124 ibid [333].

125 An appendix to this decision sets out some 40 pages of facts of the case, while there were 10,000 documents disclosed by the bank alone. ibid [3], [92] and [94].

126 ibid [396]–[397].

127
*Winnetka* (n 84) [16] (Roth J).

128
*The Ardent* (n 92) 586. Sections of the judgment interpreting the exculpatory term are at 580–1 and 586.

129 FCA, ‘Fair treatment for consumers who suffer unauthorised transactions’ (July 2015) TR15/10, [2.4] and [5.2].

130 ibid [5.1]–[5.2].

131 ibid [4.2].

132 PSD2, art 107; PSD1, art 86. PSD cases where the ECJ emphasised the maximum harmonisation goal of the legislation include *CRCAM* (n 47). The PSD is offered as an exemplar of the EU’s maximum harmonisation strategy in O Cherednychenko, ‘Islands in the Ocean: Three Models of the Relationship between EU Market Regulation and National Private Law’ (2021) 84 MLR 1294.

133 Financial Services and Markets Act 2000, Part XVI, ss 225–34B.

134 Eligible complaints and complainants are defined in the FCA Handbook, DISP 2.

135 For complaints referred after 1 April 2025 in relation to acts or omissions on or after 1 April 2019, see FOS, ‘Compensation’ <www.financial-ombudsman.org.uk/consumers/expect/compensation>.

136 FOS, ‘Financial Ombudsman Service Annual Report and Accounts for the Year Ended 31 March 2025’ 12.

137 ibid 18 and 23.

138 ibid 20.

139 ibid.

140 In relation to the APP scheme, the FOS states that it continues ‘to monitor its impact on the complaints we receive’, ibid 20.

141 HM Treasury, ‘New Approach to Ensure Regulators and Regulation Support Growth: Policy Paper’ (updated 31 March 2025) (HMT Regulatory Action Plan) <www.gov.uk/government/publications/a-new-approach-to-ensure-regulators-and-regulation-support-growth/new-approach-to-ensure-regulators-and-regulation-support-growth-html>.

142 HM Treasury, ‘Review of the Financial Ombudsman Service: Consultation’ (July 2025).

143 HMT Regulatory Action Plan (n 141).

144
*R (Williams) v FOS* [2008] EWHC 2142 [26] (Irwin J): ‘The ombudsman is dealing with complaints, not causes of action. His jurisdiction is inquisitorial not adversarial. There is a wide latitude within which the ombudsman can operate.’

145
*R (Assurant General Insurance Ltd) v FOS* [2023] EWCA Civ 1049; *R (Moniak) v FOS* [2023] EWHC 333 (Admin) [40].

146 Financial Services and Markets Act 2000, s 230A.

147 In DRN 5139910, a firm’s attempt to refer to a FOS decision in a separate case was rejected. The same point arose in DRN 2922428.

148 FCA Handbook, DISP 3.6.1, applying Financial Services and Markets Act 2000, s 228(2) (emphasis added).

149 FCA Handbook, DISP 3.6.4 (internal numbering omitted).

150
*R (Heather Moor & Edgecomb Ltd) v FOS* [2008] EWCA Civ 642 [49].

151 ibid [37]; *R (Aviva Life and Pensions (UK) Ltd) v FOS* [2017] EWHC 352 (Admin).

152 Financial Services and Markets Act 2000, s 229.

153 FOS, ‘Ombudsman Decisions’ <www.financial-ombudsman.org.uk/decisions-case-studies/ombudsman-decisions/search?IndustrySectorID[1]=1>. The searches of the FOS decisions database referenced in this section were conducted on 18 July 2025.

154 DRN 4716907.

155 DRN 4217630; on similar lines, DRN 3600048. In both cases, banks alleged that their customers had acted with gross negligence in failing to keep their devices’ security safe.

156 DRN 4355881.

157 DRN 4351159.

158 eg DRN 4775569.

159 eg DRN 4716907.

160 eg DRN 4217630.

161 eg DRN 4778679.

162 DRN 3927979.

163 DRN 3438367.

164 DRN 3764520 and DRN 8363807.

165 DRN 4063833.

166 DRN 4172461.

167 DRN 3786265.

168 DRN 3438367.

169 eg DRN 265615, where a job applicant was ‘convincingly duped’ by scammers; DRN 8363807, a ‘convincing’ phishing scam which posed as a genuine message from the bank.

170 DRN 5157757 (a corporate case); DRN 3764520.

171 DRN 4261094 and DRN 4351159. In the first, a customer was found to have fallen for an email offering vouchers which he should have been suspicious of, so fraudsters were able to access his account (no other details are provided in the short decision). In the latter, the decision reports that the customer’s version of events was unclear in places, but it was found that even after the customer had been warned that the supposed investment scheme he was ‘investing’ in was likely a scam, he continued to make payments, and he shared OTPs after warning messages from the bank. The customer was also found not to have given his bank a full account during the bank’s ‘intervention call’. Note also that this incident involved push frauds (as well as other types of payment) before the APP reimbursement scheme, so ‘gross negligence’ was considered on the basis that the PSR would have applied had the transfers been unauthorised.

172 Proposed EU Payment Regulation (n 79); Commission, ‘Proposal for a Directive on payment services in the internal market amending Directives 2002/65/EC, 2013/36/EU and 2009/110/EC and repealing Directive 2007/64/EC’ COM (2023) 547 (proposed PSD3).

173 van Praag (n 82).

174
*Heather Moor & Edgecomb Ltd* (n 150) [89] (Rix LJ).

175 van Praag (n 82) 7.2, contrasting the European Banking Authority’s suggestion for greater clarification of the term with the Council Working Party’s approach favouring a list of criteria.

176 The extent to which the UK government decides that it is in the country’s interests to reform UK regulation to be equivalent to the EU’s is a separate point, outside the scope of this article.

177 European Parliament legislative resolution of 23 April 2024 on the proposal for a regulation on payment services in the internal market and amending Regulation (EU) No 1093/2010 (COM(2023)0367—C9-0217/2023—2023/0210(COD)), proposed Recital 82a.

178 van Praag (n 82) 7.2.

179 ibid 7.2: drawing on Dutch case law, the EBI working paper offers a suggested definition as follows: ‘a serious degree of culpability on the part of the [customer] and that, in order to establish gross negligence, the PSP must prove that the user was aware or should have been aware under the concrete circumstances of a specific risk of becoming victim of payment fraud’ ibid, 7.2 (footnotes omitted).

180 ibid 7.2.

181
*Philipp* (n 10) [23] (Lord Leggatt).

182 van Praag (n 82) 14.

183 See s 2B above; Banking Code (n 20) para 1.4.

184 Examples of FOS decisions which were required to determine whether a payment was authorised or unauthorised or where there was confusion on this point include DRN 4793731, DRN 509023, DRN 5018566, DRN 4716907 and DRN 4775569.

185 van Praag (n 82) s 6.2.

